# Preventable Burn Injury in a Person With Paraparesis Leading to Amputation: A Case Report

**DOI:** 10.7759/cureus.79526

**Published:** 2025-02-23

**Authors:** Abhimanyu Vasudeva, Suryakanta Seth

**Affiliations:** 1 Physical Medicine and Rehabilitation, All India Institute of Medical Sciences, Gorakhpur, IND; 2 Anatomy, All India Institute of Medical Sciences, Gorakhpur, IND

**Keywords:** burns, pressure ulcer, quality of life, sensation, spinal cord injuries

## Abstract

Spinal cord injury (SCI) often results in prolonged recovery and complications, as seen in the case of a 35-year-old mason who sustained an SCI after a 10-foot fall in June 2021. Initially presenting with lower limb paresis and loss of bladder and bowel control, he underwent D12-L2 fixation with decompression and showed a gradual recovery of motor and sensory function above the knees. However, due to sensory loss and inadequate awareness regarding pressure injury prevention, he developed sacral pressure sores, which were healing with appropriate wound care and pressure offloading. During this period, he sustained a severe burn to his right lower limb from accidental contact with a hot bike exhaust, which went unnoticed due to impaired sensation. Despite conservative treatment, the burn injury progressed to neuropathic ulcer formation, Charcot arthropathy, and secondary infection with osteomyelitis, necessitating repeated pus aspirations. Over six months, the infection persisted despite multiple hospital visits and empiric antibiotic therapy. Given the chronicity, repeated aspirations, and ongoing treatment burden, he was offered the option of a below-knee amputation as a definitive treatment after a thorough discussion of risks, benefits, and alternatives. Following amputation, he is planned for prosthetic fitting and comprehensive rehabilitation. This case highlights the importance of timely intervention, awareness of pressure injuries, and proactive management of sensory deficits to prevent secondary complications such as infections and amputations. A multidisciplinary approach, patient education, and preventive strategies are essential to improving outcomes and quality of life in individuals with SCI.

## Introduction

Spinal cord injury (SCI) leads to motor and sensory impairment, significantly increasing the susceptibility to pressure injuries [1]. As people with spinal cord injuries engage in diverse activities, they may encounter new risks, including burn injuries due to sensory loss [2].

Pressure ulcers are a well-recognized complication in individuals with chronic SCI, with a reported prevalence ranging from 15% to 30%. Despite the availability of various risk assessment tools, most have been developed based on general pathophysiological principles rather than being specific to SCI-related vulnerabilities. In recent years, there has been an effort to create an SCI-specific risk assessment model, but initial findings suggest limitations in accurately predicting pressure ulcer development due to variability in the level of evidence supporting different risk factors.

The risk factors for pressure ulcer development in individuals with chronic SCI can be categorized into sociodemographic, neurological, medical, and behavioral factors. While sociodemographic and neurological factors have a relatively high level of evidence, behavioral factors, such as pressure-relief practices, routine skin monitoring, and smoking, show lower levels of supporting evidence. However, these behavioral factors are potentially modifiable, making them important targets for preventive strategies and therapeutic education programs aimed at reducing pressure ulcer incidence. Developing more specific assessment tools to evaluate these behavioral risk factors will be crucial in improving early identification and prevention of pressure ulcers in individuals with SCI [3].

Individuals with SCI are at an increased risk of secondary complications, including pressure ulcers and unintentional injuries due to impaired sensation. One such preventable injury is burns. In this case, a 35-year-old mason who sustained an SCI following a fall from a height in June 2021 developed sacral pressure sores due to prolonged immobility. He sustained a severe thermal burn on his right lower limb after unknowingly resting it against a hot bike exhaust. Due to impaired sensation, the injury was not immediately recognized and subsequently progressed over time. Despite standard wound care measures, the burn evolved into a chronic neuropathic ulcer, eventually leading to Charcot arthropathy and secondary infection. Persistent infection and the need for repeated interventions over six months ultimately led to the consideration of below-knee amputation as a definitive treatment.

Thorough pre-surgical seating evaluation, individualized postsurgical rehabilitation, and regular follow-up with seating adjustments can help prevent recurrent pressure injuries in individuals with spinal cord injuries [4]. A study by Recio et al. highlights the functional and psychological benefits of prosthetic use in a person with thoracic paraplegia post-amputation [5].

The objective of this case report is to highlight how insufficient preventive care led to the avoidable amputation of a lower limb in an SCI patient. This case report aims to emphasize the importance of early rehabilitation, proper management of pressure injuries, and patient awareness of sensory loss as part of a holistic approach.

A preventable burn injury, compounded by impaired sensation and delayed recognition, led to complications that eventually necessitated a below-knee amputation. While preventive care measures, including patient education on sensory deficits and strategies to avoid inadvertent injuries, are integral components of standard SCI management, challenges such as limited awareness, resource constraints, and variations in access to specialized care may impact their implementation.

The standard of care emphasizes comprehensive preventive strategies, including early rehabilitation, regular skin monitoring, and patient education to mitigate risks. However, despite best efforts, certain complications may still arise due to multifactorial influences. This case highlights the importance of reinforcing preventive measures and ensuring timely interventions to optimize outcomes for individuals with SCI.

The burn injury occurred before the patient reached us; therefore, it is unlikely that the patient received preventive instructions beforehand, as also communicated during our discussion. Preventive education and injury avoidance strategies are part of the standard of care in spinal centers globally. However, in this case, the patient sustained the burn injury in the community setting before specialized care could be initiated.

This case highlights the critical need for early and widespread patient education on sensory deficits and injury prevention, particularly for individuals with newly acquired SCI who may not yet be aware of their vulnerability to such injuries.

Medically underserved individuals with SCI remain vulnerable to severe pressure injuries despite preventive education. A study analyzing treatment notes from a randomized controlled trial (RCT) examined 25 community-dwelling adults with SCI who developed 40 serious pressure injuries. The key contributing factors included limited wound care knowledge, inadequate equipment, comorbidities, non-adherence to bed rest, inactivity, and external constraints. Addressing these challenges through patient-specific education, health literacy assessment, and improved access to medical equipment may enhance rehabilitation outcomes [6].

## Case presentation

The patient, a 35-year-old male mason with no significant past health concerns, was in satisfactory overall health before sustaining a fall from a height in the year 2021. His profession entailed physical labor, frequently at building sites. While working, he fell from a height of 10 feet from a temporary structure. Post-fall, he was unable to get up. As the injury occurred long before the patient's presentation to our facility, we do not have firsthand findings. The available documentation does not explicitly mention these details, and in the absence of concrete information, we prefer to refrain from making speculative comments. Based on the available history, muscle power was estimated to be less than 3/5, according to the Medical Research Council (MRC) grading scale.

An MRI of the spine conducted in June 2021 revealed a compression fracture of the L1 vertebral body with retropulsion of the posterior vertebral margin, causing impingement of the thecal sac, the tip of the conus medullaris, and root of the cauda equina. T2 hyperintensity was noted in the cord parenchyma, suggestive of compressive/traumatic myelopathy, along with paraspinal soft tissue edema. The patient subsequently underwent D12-L2 fixation with decompression. Given the involvement, the recovery has a variable prognosis, with some patients showing improvement over time and others experiencing persistent deficits.

**Figure 1:**
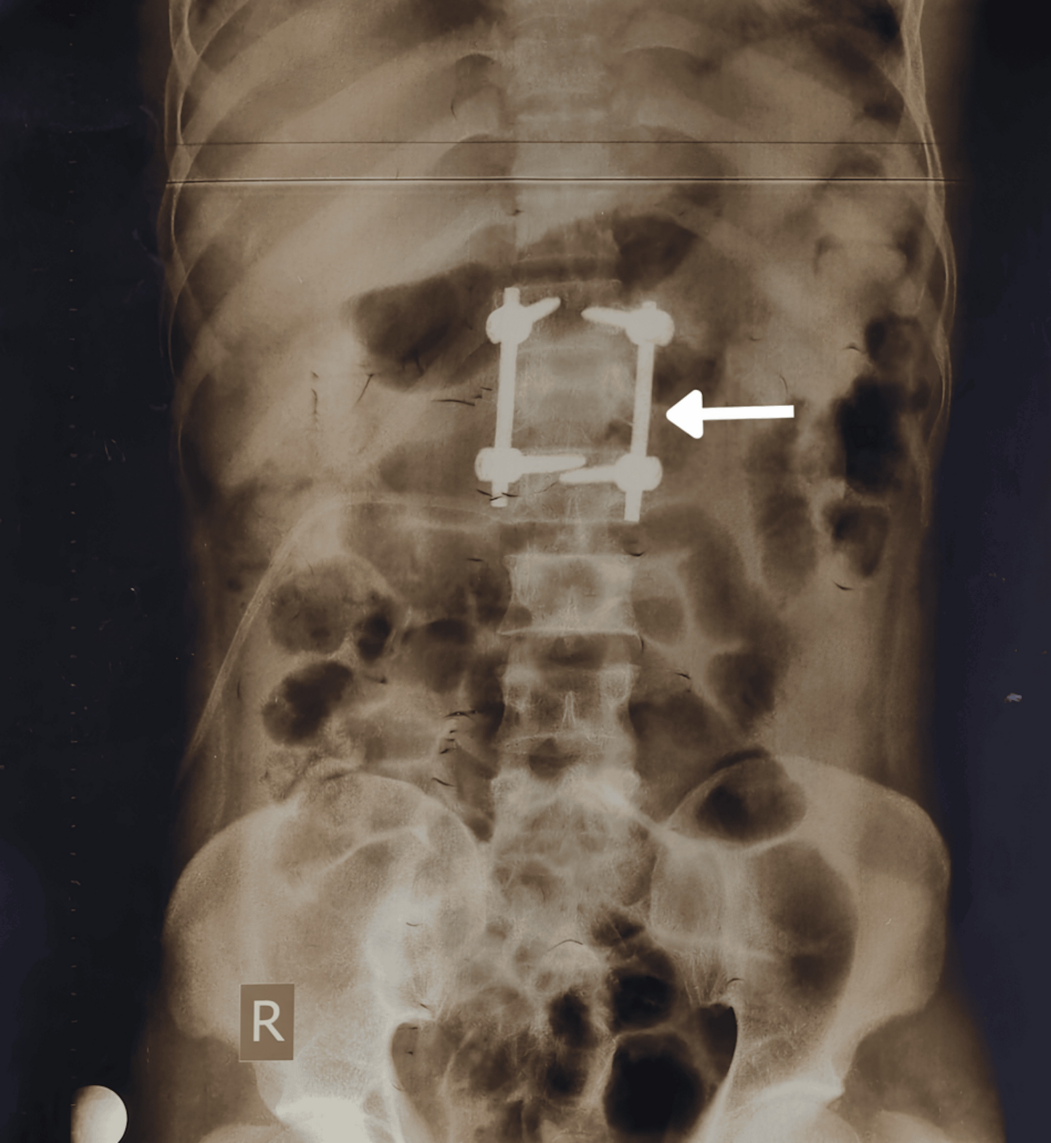
A radiograph obtained prior to his presentation with the burn injury showing spinal hardware (white arrow) indicative of previous spinal surgery.

The radiographic imaging illustrates the surgical instrumentation in this case (Figure 1).

Upon discharge, he had not regained strength in his lower limbs or control over his bladder and bowel. He continued with indwelling catheterization for another month, with changes every 15 days. Bladder and bowel control began to improve approximately two months post-injury, with complete control regained after another two months. He also noted mild improvement in lower limb strength. He was advised by the hospital to start walking with manual support and gradually began sitting and walking with a walker over the next two to three months. The patient initially received care at another facility before presenting to our center. The term manual support refers to assistance provided by another person to help the patient with balance and movement during early mobilization.

At our center, routine care for post-spinal injury patients involves a comprehensive approach tailored to the patient's neurological status and functional goals. Initial management includes a thorough neurological evaluation to determine the level and severity of the injury, followed by preventive measures such as education regarding proper precautions related to the skin, frequent repositioning, and specialized support surfaces to minimize the risk of pressure ulcers. Bladder and bowel management is a key component, utilizing intermittent catheterization or other appropriate methods alongside dietary modifications and medications as needed. Rehabilitation focuses on gradual mobilization, beginning with assisted sitting, balance training, and strength exercises, eventually progressing to gait training with appropriate assistive devices based on neurological recovery. Spasticity and pain are managed through a combination of medications and therapy, ensuring optimal comfort and mobility.

Additionally, psychosocial support, patient education, and vocational rehabilitation play a crucial role in helping individuals reintegrate into their communities and regain independence. A neurological examination was done as per protocol at periodic visits. Given the complex nature of SCI, sensory recovery may not always correlate precisely with the site of surgical intervention. The sensory recovery above the knee was more or less in line with motor, bladder, and bowel recovery timelines. However, he did not regain sensation below the knee and in the sacral region. Approximately three months post-injury, he developed pressure injuries, including three sacral bedsores. Despite regaining some ambulatory function with a walker, the patient's sensory impairment and impaired pressure relief mechanisms likely contributed to pressure injuries. As initial management occurred elsewhere, we cannot confirm the preventive measures taken at that stage. However, it appears that it was not emphasized based on the conversation with the patient.

**Figure 2:**
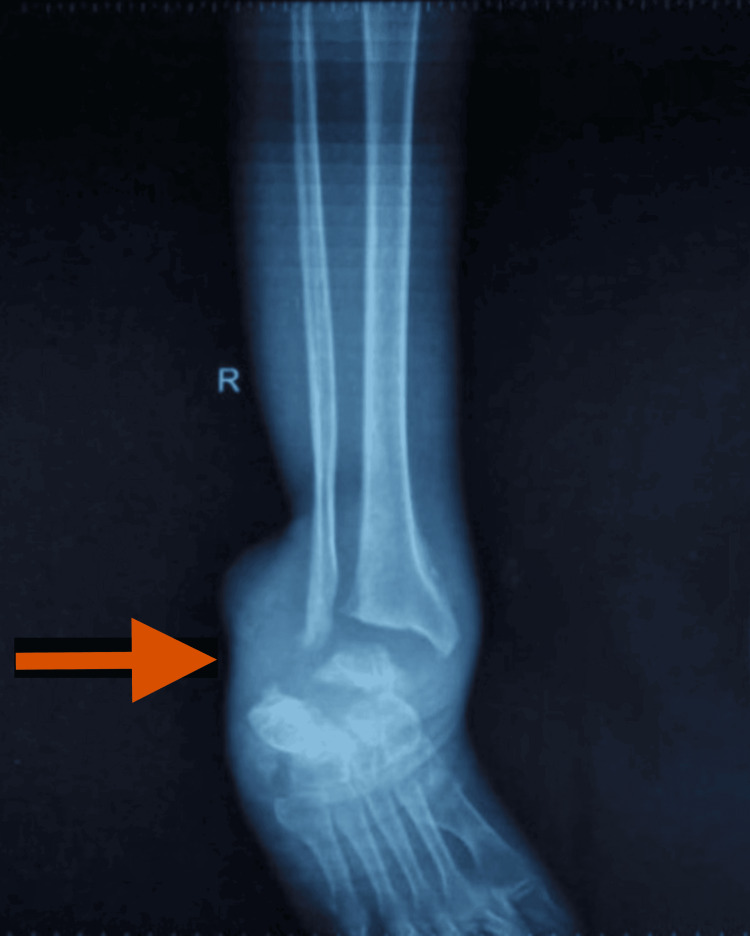
The radiograph shows bony destruction and deformity (arrow).

Prior to the injury, the patient was fully independent in ambulation and activities of daily living, with no documented neurological or functional deficits. Two years post-injury, while traveling as a pillion rider on a bike, he sustained burns to his right lower limb from the exhaust. At this stage, the patient visited the Department of General Surgery and the Department of Physical Medicine and Rehabilitation at an Institute of National Importance in Northern India for further evaluation and management of the case. He was treated for bedsores and evaluated for the burnt lower limb. An attempt to heal the burn injuries using medications, including dressings, was made. The patient sustained a severe burn on his right lower limb due to accidental contact with a hot bike exhaust, which remained unnoticed because of impaired sensation. Despite conservative treatment, the burn progressed to a neuropathic ulcer, eventually leading to Charcot arthropathy and secondary infection with osteomyelitis. Repeated pus aspirations were required, but the infection persisted over six months despite multiple hospital visits and empiric antibiotic therapy. Given the chronic nature of the condition, ongoing infections, and repeated interventions, he was offered the option of a below-knee amputation as a definitive treatment after a comprehensive discussion of risks, benefits, and alternatives (Figure 2).

**Figure 3:**
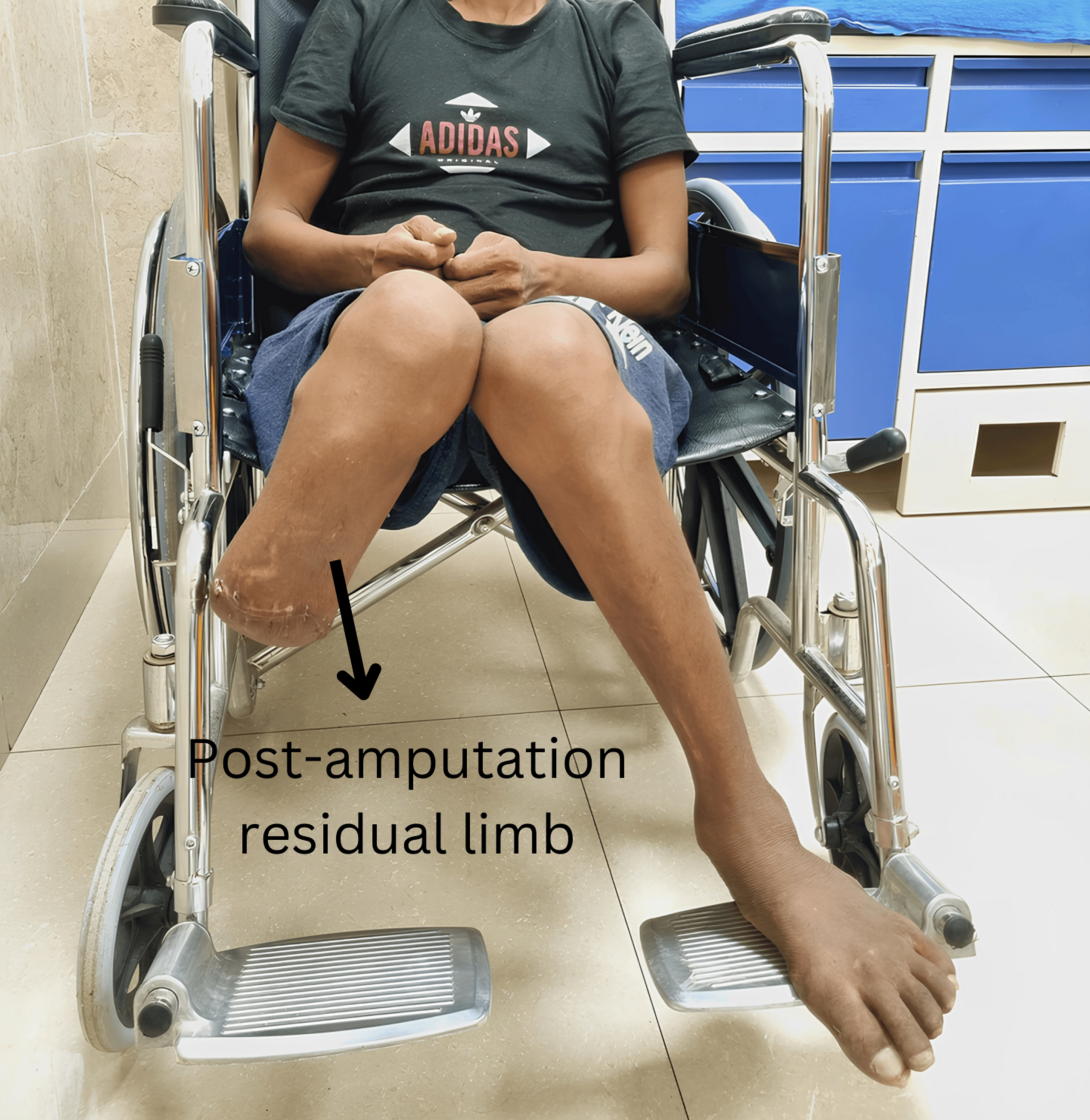
Post-amputation residual limb.

When conservative management and repeated interventions failed to control the infection and preserve limb function, a below-knee amputation was performed to prevent further complications and improve overall health and mobility (Figure 3).

He was then referred for a prosthesis. Given the insensate nature of the region below the knee, a lot of consideration and discussion with the patient occurred before prescribing a prosthesis. A silicon suction socket with proper pressure mapping, an endoskeletal shank, and a SACH (solid ankle-cushion-heel) foot were discussed as an option, acknowledging the risk of injury to the residual limb. Rehabilitation included range of motion and stretching exercises, particularly for the hip and knee flexors. The prosthesis has been planned to be designed to accommodate his limited ambulation needs, primarily for household use with a walker. His wife works on the farm and earns the family's livelihood. He receives social security benefits as per the law.

Informed written consent was obtained from the patient prior to publication, ensuring their understanding of the content and voluntary participation while adhering to ethical guidelines and privacy protocols.

## Discussion

This case highlights that even a seemingly minor burn injury can progress to severe complications, leading to the need for drastic measures such as amputation. The patient's right lower limb injury, caused by accidental contact with a hot bike exhaust, exemplifies how an unnoticed injury, due to impaired sensation, can evolve into a neuropathic ulcer and eventually Charcot arthropathy and secondary infection with osteomyelitis. This progression highlights the importance of proactive, timely interventions and preventive measures, particularly in individuals with SCI.

Interventions and preventive care are essential for reducing secondary complications in SCI patients. The optimal surgical approach for SCI remains under active investigation [7]. Multifaceted prevention strategies, including education and improved nursing practices, have been effective in reducing hospital-acquired pressure ulcers and enhancing patient outcomes. Further research is needed to optimize interventions, evaluate long-term impact, and establish standardized protocols across healthcare settings [8].

Effective prevention of pressure ulcers in individuals with SCI requires continuous education, customized interventions, and access to specialized care, ensuring that post-discharge needs align with best practices to reduce complications and improve quality of life [9]. Telemedicine has proven to be a feasible and cost-effective approach to preventing pressure injuries in individuals with spinal cord injuries, especially when used alongside other preventive measures. Its ability to lower the risk of these injuries without adding financial strain highlights the importance of further research to enhance its implementation and effectiveness [10].

Enhancing rehabilitation through collaboration between healthcare professionals and operations researchers has shown significant potential in improving service quality and patient-centered care, aligning with recent global initiatives by the World Health Organization (WHO) [11]. An effective approach to preventing pressure ulcers must consider the interplay between individual lifestyle factors, external environmental influences, and the healthcare system's impact on patient behavior and adherence to preventive measures [12].

This case emphasizes the critical role of early intervention, comprehensive rehabilitation, and a multidisciplinary approach in reducing long-term complications for patients with SCIs. Without timely management, conditions such as pressure ulcers can progress to severe, avoidable health issues. Structured rehabilitation programs, patient education, and consistent monitoring are essential in preventing these outcomes. Evidence suggests that telemedicine has improved chronic disease management by expanding patient access, enabling remote monitoring, and lowering healthcare costs. Applying similar telehealth strategies in SCI care could help prevent complications such as pressure injuries, infections, and lapses in follow-up care, ultimately improving patient outcomes [13].

It is essential to educate new graduates and facilitate the dissemination of knowledge and skills related to rehabilitation [14].

The patient's perspective was sought, and he expressed distress over the amputation, stating he had not been sufficiently informed about the risks of burns and pressure injuries due to sensory impairment. While initially unsure about using a prosthesis, he was hopeful about restoring mobility and adjusting to daily activities. His main objective was to regain independence and assist his wife, who had become the primary earner. He felt that greater awareness of injury prevention might have prevented the amputation and hoped his experience would help others in similar situations.

## Conclusions

This case highlights the severe consequences of unrecognized injuries in individuals with SCI, emphasizing the necessity of early detection and preventive strategies. The progression of an unnoticed burn injury to chronic infection and eventual lower limb amputation highlights the critical role of patient education, sensory monitoring, and timely intervention. Comprehensive rehabilitation, including structured awareness training, pressure injury prevention, and regular follow-up, is essential to mitigate such risks. By prioritizing early recognition of sensory deficits and proactive injury prevention, healthcare providers can significantly reduce the likelihood of avoidable complications and improve patient outcomes.
